# Culprit or Bystander: Defective Mitophagy in Alzheimer’s Disease

**DOI:** 10.3389/fcell.2019.00391

**Published:** 2020-01-17

**Authors:** Chenglong Xie, Yahyah Aman, Bryan A. Adriaanse, M. Zameel Cader, Hélène Plun-Favreau, Jian Xiao, Evandro F. Fang

**Affiliations:** ^1^Department of Neurology, The First Affiliated Hospital of Wenzhou Medical University, Wenzhou, China; ^2^Department of Clinical Molecular Biology, University of Oslo, Akershus University Hospital, Lørenskog, Norway; ^3^Weatherall Institute of Molecular Medicine, University of Oxford, Oxford, United Kingdom; ^4^Queen Square Multiple Sclerosis Centre, Department of Molecular Neuroscience, UCL Queen Square Institute of Neurology, University College London, London, United Kingdom; ^5^Molecular Pharmacology Research Center, School of Pharmaceutical Sciences, Wenzhou Medical University, Wenzhou, China; ^6^The Norwegian Centre on Healthy Ageing (NO-Age), Oslo, Norway

**Keywords:** Alzheimer’s disease, mitophagy, aging, neuroprotection, memory

## Abstract

Mitophagy is a selective engulfment and degradation of damaged mitochondria through the cellular autophagy machinery, a major mechanism responsible for mitochondrial quality control. Increased accumulation of damaged mitochondria in the Alzheimer’s disease (AD) human brain are evident, although underlying mechanisms largely elusive. Recent studies indicate impaired mitophagy may contribute to the accumulation of damaged mitochondria in cross-species AD animal models and in AD patient iPSC-derived neurons. Studies from AD highlight feed-forward vicious cycles between defective mitophagy, and the principal AD pathological hallmarks, including amyloid-β plaques, tau tangles, and inflammation. The concomitant and intertwined connections among those hallmarks of AD and the absence of a real humanized AD rodent model present a challenge on how to determine if defective mitophagy is an early event preceding and causal of Tau/Aβ proteinopathies. Whilst further studies are required to understand these relationships, targeting defective mitophagy holds promise as a new therapeutic strategy for AD.

## Molecular Mechanisms of Mitophagy and Its Roles in Neuroplasticity

Mitophagy is a highly conserved cellular process of removing damaged or superfluous mitochondria to maintain mitochondrial homeostasis (Pickrell and Youle, 2015; Scheibye-Knudsen et al., 2015; Fang et al., 2016b; McWilliams et al., 2016; Fivenson et al., 2017). In neurons, accumulation of damaged mitochondria is noxious to cellular function and survival. Mitophagy, at physiological level, maintains neuroplasticity and the functions of glial cells (Gustafsson and Dorn, 2019). Recent findings in human cell lines and multiple animal models have extended our knowledge in the molecular mechanisms of mitophagy from the PINK1-Parkin pathway, to the PINK1-independent pathways, including pathways that depend on NIP3-like protein X (NIX), B-cell lymphoma 2 interacting protein 3 (BNIP3), B-cell lymphoma 2-like 13 (BCL2L13), FK506 binding protein 8 (FKBP8), prohibitin (PHB2), breast cancer gene 1 protein (NBR1), optineurin (OPTN), calcium binding and coiled-coil domain 2 (NDP52), Autophagy and Beclin 1 Regulator 1 (AMBRA1), Tax1 binding protein 1 (TAX1BP1), FUN14 domain-containing protein 1 (FUNDC1), PGAM family member 5 (PGAM5), Nipsnap Homolog 1 (NIPSNAP1), NIPSNAP2, among others (Fivenson et al., 2017; Kerr et al., 2017; Palikaras et al., 2018; Lou et al., 2019; Princely Abudu et al., 2019).

The PINK-1-dependent mitophagy is one of the well-characterized mitophagy pathways, with mutations of *PINK1* associated to familial Parkinson’s disease (PD) (Plun-Favreau and Hardy, 2008; Gandhi et al., 2009; Burchell et al., 2013; Pickrell and Youle, 2015). Under physiological conditions, mitochondrial membrane potential (MMP) drives mitochondrial import of the 63 kDa full length PINK1. Presenilin-associated rhomboid-like protein (PARL) is an inner mitochondrial membrane (IMM) protease. PARL cuts the mitochondrial targeting sequence (MTS) and trans-membrane domain of PINK1, leading to the cytosolic release of the N-terminal-deleted PINK1 (ΔN-PINK1) (Deas et al., 2011). The N-terminal-deleted PINK1 (ΔN-PINK1) is degraded by the N-end rule pathway and the ubiquitin proteasome system (Pickrell and Youle, 2015). However, under various stressors or MMP fluctuations, PINK1 is shunted and retained on the outer mitochondrial membrane (OMM), promoting Parkin recruitment to the defective mitochondrial surface with the help of PINK1 autophosphorylation (Hasson et al., 2013; Lazarou et al., 2015). Parkin, an E3 ubiquitin ligase, ubiquitinates several OMM proteins, including voltage-dependent anion-selective channel protein (VDAC), mitofusin 2 (Mfn2), and dynamin-1-like protein (DRP1), leading to their recognition by autophagic adaptors: OPTN, NDP52, sequestosome 1 (SQSTM1/p62), TAX1BP1, or NBR1 (Sarraf et al., 2013; Lazarou et al., 2015; Ordureau et al., 2018).

**FIGURE 1:**
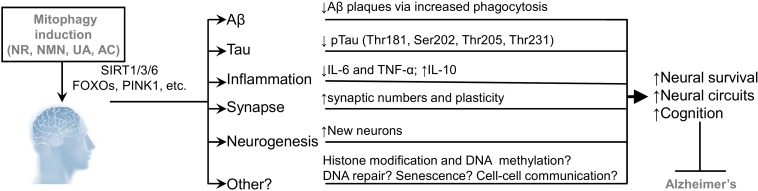
A summary of possible molecular mechanisms on how mitophagy induction ameliorates AD pathologies. Experimental studies from *C. elegans* and mouse models of AD and from AD iPSC-derived neurons indicate genetic or pharmacological up-regulation of mitophagy inhibits Aβ/Tau proteinopathies and inflammation, as well as promotes synaptic plasticity and neurogenesis. Robust health-benefit mitophagy inducers include the NAD^+^ precursors, nicotinamide riboside (NR) and nicotinamide mononucleotide (NMN), urolithin A (UA), and actinonin (AC). NAD^+^ augmentation activates the NAD^+^-dependent SIRT1/3/6 activities, and increases the expression and/or activities of autophagic/mitophagic proteins, including LC3-II and PINK1 and NIX, among others. Whether mitophagy induction improves histone modification and DNA methylation, neuronal DNA repair, cell-to-cell communication, and limits senescence remain to be determined. See text for more details as well as references.

Growing evidence indicates the existence and importance of PINK1- and/or Parkin-independent pathways. In addition to Parkin, other E3 ubiquitin ligases, such as mitochondrial ubiquitin ligase activator of NF-kB1 (MUL1), seven *in absentia* homolog 1 (SIAH1), Gp78, SMAD ubiquitin regulatory factor 1 (SMURF1), and Ariadne RBR E3 ubiquitin protein ligase 1 (ARIH1) participate in mitophagy. These E3 ubiquitin ligases are localized on OMM to generate ubiquitin chains, in order to direct coupling to the autophagy protein LC3, enabling the engulfment of the ubiquitin chain-tagged mitochondria by phagosomes, and finally fusion with the acidic lysosome to degrade the damaged mitochondria (Szargel et al., 2016; Villa et al., 2017). In addition to ubiquitin ligase-dependent mitophagy, OMM proteins can act as mitophagy receptors, targeting damaged mitochondria directly for mitophagy-mediated degradation. Examples include: BNIP3, NIX/BNIP3L, and FUNDC1 that mediate mitochondrial elimination via display of the N-terminus LIR domain into the cytosol which interact with LC3 or gamma-aminobutyric acid receptor-associated protein (GABARAP) (Sandoval et al., 2008; Liu et al., 2012; Zhang et al., 2016; Palikaras et al., 2018; Villa et al., 2018; Lou et al., 2019). Additionally, PHB2 and cardiolipin are amongst the recently discovered mitophagy proteins, which can externalized to OMM and couple with LC3 following mitochondrial membrane depolarization (Shen et al., 2017; Wei et al., 2017). In summary, while the PINK1/Parkin-dependent mitophagy pathway is well-characterized, the molecular mechanisms of multiple new mitophagy pathways are still not fully understood (Figure 1).

## Defective Mitophagy in AD

Whilst accumulated extracellular Aβ plaques and intraneuronal Tau tangles are the disease-defining pathological features of Alzheimer’s disease (AD), inflammation is now widely recognized as a key additional hallmark of AD. Relationships between mitophagy and each of the hallmarks of AD are summarized below.

### Mitophagy and Amyloid-β (Aβ)

Neurons affected in AD models undergo defective mitophagy that contribute to the disease-defining Aβ pathologies, while Aβ accumulation may exacerbate impaired mitophagy and *vice versa* (Du et al., 2017; Kerr et al., 2017; Fang, 2019; Fang et al., 2019). Impaired mitochondrial proteostasis, including impaired mitochondrial unfolded protein response (UPR^*mt*^), may link to Aβ proteotoxicity (Sorrentino et al., 2017). The activating transcription factor-associated with stress (ATFS-1) protein plays a fundamental role in the maintenance of UPR^*mt*^ and mitochondrial function, especially in stress conditions (Nargund et al., 2012). RNAi knockdown of *atfs-1* in an Aβ *Caenorhabditis elegans* model (GMC101) repressed mitophagy as well as basal and maximal respiration, and exacerbated Aβ toxicity; However, restoration of UPR^*mt*^ diminished AD pathology in both *C. elegans* and mouse models of AD (Sorrentino et al., 2017). Mechanistically, ATFS-1 transfers into and is degraded within mitochondrial matrix, which negatively impacts UPR^*mt*^, at physiological condition (Melber and Haynes, 2018). Under the condition of mitochondrial stress, ATFS-1 favors importation into the nucleus, whereby it promotes the expression of genes with encoded proteins involved in the protection of mitochondrial function and the elimination of AD pathology (Melber and Haynes, 2018). In support of this model, mutations that cause amino acid substitutions within the MTS of ATFS-1 prevent the protein from being imported into the mitochondrial matrix, and result in constitutive UPR^*mt*^ activation (Rauthan et al., 2013). Abnormal mitochondrial homeostasis was reported in the mutant APP-HT22 cells relative to non-transfected HT22 cells, including increased levels of mitochondrial fission proteins (Drp1 and Fis1) and decreased levels of fusion proteins (Mfn1, Mfn2, and Opa1) (Manczak et al., 2018; Reddy et al., 2018). In addition to impaired UPR^*mt*^, defective mitophagy is another major cause of impaired mitochondrial proteostasis and Aβ proteinopathy in AD. On one hand, defective mitophagy in post-mortem brain tissues from AD patients as well as in AD iPSC-derived neurons and cross-species Aβ-based AD animal models have been demonstrated (Fang et al., 2019). On the other, restoration of neuronal and microglial mitophagy ameliorated Aβ proteinopathy and rescued memory loss in the APP/PS1 mouse models of AD, highlighting the important contribution of defective mitophagy in AD (Fang et al., 2019). Disrupted−in−schizophrenia−1 (DISC1), an LC3-binding mitophagy protein, has been shown to be reduced in human AD brain samples and in the APP/PS1 mice. In fact, Aβ−induced mitochondrial dysfunction, loss of spines, and impaired long−term potentiation (LTP) were rescued upon DISC1 overexpression in the APP/PS1 mice (Wang et al., 2019). Collectively, the current studies implicate that impaired mitochondrial proteostasis as a contributor to Aβ-based neurotoxicity via impaired UPR^*mt*^ and compromised mitophagy. However, the detailed molecular mechanisms remain to be determined.

### Mitophagy and Tau

Tau binds and stabilizes microtubules, contributing in multiple physiological functions, such as neurite outgrowth, neuronal development, axonal transport, and synaptogenesis (Ballatore et al., 2007; Dixit et al., 2008). Studies in experimental AD models have provided evidence that mitochondrial dysfunction, defective mitophagy and phosphorylated-Tau (p-Tau) interact to form a vicious cycle (Kerr et al., 2017). The toxic N-terminal truncation of human Tau (NH_2_-hTau) strongly affects the interplay between the mitochondria dynamics and mitophagy affecting subcellular trafficking or recruitment of both Parkin and ubiquitin-C-terminal hydrolase L1 (UCHL-1) (Amadoro et al., 2014; Corsetti et al., 2015). In *C. elegans* and neuroblastoma cells, expression of human wild-type (hTau) and frontotemporal dementia mutant tau (hP301L) completely inhibited mitophagy by blocking the recruitment of Parkin to damaged mitochondria (Cummins et al., 2019). Furthermore, APP and tau overexpression lead mitophagy impairment in human unmodified fibroblasts (Martin-Maestro et al., 2019). Furthermore, mitophagy was impaired in hippocampus tissues from 3xTgAD mice (with both Aβ and Tau proteinopathies) (Fang et al., 2019). In addition, pharmacological restoration of mitophagy, via administration of NAD^+^ precursor nicotinamide mononucleotide (NMN), urolithin A (UA), or actinonin (AC), reduced the phosphorylation of pTau at several sites (such as Thr181, Ser202/Thr205, Thr231, and Ser262) (Fang et al., 2019). Collectively, emerging evidence suggests that pathological Tau inhibits mitophagy, highlighting defective mitophagy as a novel therapeutic target for AD.

### Mitophagy and Inflammation

Numerous preclinical and clinical studies have shown that immune activation in AD, including microglia, and several cytokines, has the capacity to trigger and drive the pathophysiology of AD (Heppner et al., 2015). Mitochondrial stress leads to the release of damage-associated molecular patterns (DAMPs) which activate innate immunity, with the Cyclic GMP-AMP synthase (cGAS)-STING pathway as a central regulator of the type I interferon response to cytosolic DNA (Ishikawa and Barber, 2008; Ishikawa et al., 2009; Chen et al., 2016). Mitophagy mitigates inflammation through the restriction of inflammatory cytokine secretion and the regulation of immune cell homeostasis, correlating with the pathogenesis of autoimmune diseases at multiple levels (Xu et al., 2019). Multiple studies have demonstrated that PINK1 and Parkin regulate both innate and adaptive immunities. First of all, there is a strong inflammatory phenotype in both *Pink1^–/–^* and *Parkin^–/–^* mice, both of which were central regulators in the mitophagy process. Furthermore, PINK1 and Parkin mitigated STING-induced inflammation and rescued the loss of dopaminergic neurons from the substantia nigra (SN) in both *Pink1^–/–^* and *Parkin^–/–^* mice following exhaustive exercise (Sliter et al., 2018). Additionally, PINK1 and Parkin regulate immunity by repressing mitochondrial antigen presentation (MitAP) via mitochondria-derived vesicles (MDVs) (Matheoud et al., 2016). While the roles of STING and MitAP in the inflammation phenotype of AD is obscure, impairment of the PINK1/Parkin pathway in AD (Sliter et al., 2018; Fang et al., 2019), points to a possibility of an overlapping effect between PD and AD. The concomitant and intertwined molecular pathways that link defective mitophagy to Aβ and Tau proteinopathies, and inflammation need further exploration. Lastly, restoration of neuronal mitophagy (through NAD^+^ supplementation, UA, and AC) reduced AD pathologies in the APP/PS1 AD mice via enhanced microglial phagocytosis of extracellular Aβ plaques and the mitigation of pro-inflammatory cytokines released by continually activated microglia (Fang et al., 2019). Changes of mitophagy in AD astrocytes are elusive. It has been show that astrocytes play an important role in mitophagic degradation of damaged mitochondria from adjacent neurons (Davis et al., 2014), thus mitophagy induction may also improve different functions of astrocytes in AD. A recent development of a three-dimensional (3D) human AD triculture model, with neurons, astrocytes, and microglia (Park et al., 2018), may enable the studies of cell type-specific mitophagy in an environment which mimic the human brain. Collectively, while defective mitophagy plays a pivotal role in AD progression, and turning up mitophagy forestalls AD pathology, further molecular mechanisms on how mitophagy induction impacts neurons, astrocytes and microglia are necessary.

## Defective Mitophagy in Other Neurodegenerative Disease

PD is a progressive neurological disorder that observably impairs patients’ ability to control body balance and movements due to lack of dopaminergic neurons in the substantia nigra (SN), which exhibits abnormal accumulation of α-synuclein fibrils in their cell body and neurites (Poewe et al., 2017). Mitochondrial dysfunction and its related oxidative stress and inflammation are increasingly appreciated as common features of dopaminergic neuronal susceptibility in PD patient brain samples, PD animal models, and/or PD iPSC-derived neurons (Ryan et al., 2015; Schondorf et al., 2018). As a classical mitophagy pathway, the PINK1/Parkin pathway eliminates damaged mitochondria. Loss-of-function mutations in *PINK1* and/or *PARK2/Parkin* lead to inability of the cell to eliminate damaged mitochondria, and this has been related to early onset PD (Ryan et al., 2015). In addition, PINK1 and Parkin also suppress mitochondrial antigen presentation (MitAP) probably through inhibition of Sorting nexin 9 (Snx9)-dependent formation of MDVs (Matheoud et al., 2016). Meanwhile, *Parkin*- and *Pink1*-mutant fly models recapitulate major phenotypes of PD, including mitochondrial dysfunction, dopaminergic neuronal loss, motor disabilities and reduced lifespan (Yang et al., 2006). For mice, while the *Parkin^–/–^* and *Pink1^–/–^* animals do not show PD phenotypes at standard laboratory living condition, they do exhibit PD phenotypes (e.g., the loss of dopaminergic neurons) at stress living conditions, such as intestinal infection, exhaustive exercise, and mitochondrial stress (Perez and Palmiter, 2005; McWilliams et al., 2018; Sliter et al., 2018; Matheoud et al., 2019). These rodent data suggest compensation of the loss of PINK1-dependent mitophagy by PINK1-independent pathways under physiological conditions are sufficient; however, the PINK1-pathway is necessary at stress/pathological conditions for the function and survival of PD-related dopaminergic neurons.

**FIGURE 2:**
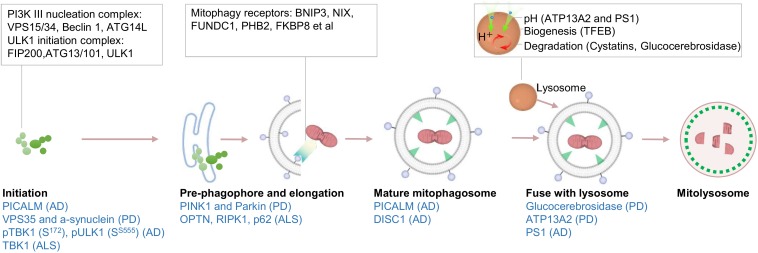
Schematic of mitophagy pathway and its linkages to different neurodegenerative diseases. A simplified version of mitophagy is presented. Initiation of mitophagy is activated via the activity of the ULK1 complex and PI3K complex. Precursor vesicles fuse to form pre-phagophore structures that further elongate to eventually become double-membraned mitophagosomes. The completed autophagosomes are then trafficked to fuse with lysosomes to form mitolysosome. Dysfunction throughout this pathway, from initiation of mitophagosome formation to degradation in the mitolysosomes, have been suggested to be involved in AD, PD, and ALS (marked in blue).

Amyotrophic lateral sclerosis (ALS) is a fatal neurodegenerative disease (predominately sporadic, nearly 90%) characterized by the accumulation of aggregated proteins partially resulted from mitochondria dysfunction and oxidative stress within affected motor neurons in the spinal cord, brain stem, and motor cortex (Rowland and Shneider, 2001). Genetic studies of familial ALS have identified several genes linked to ALS (Cirulli et al., 2015). Most of the genes involved in cellular quality control pathways, and more specifically to selective autophagy and mitophagy, including mitophagy receptors OPTN, RIPK1, p62/SQSTM1, as well as TBK1 (Cirulli et al., 2015; Hawk et al., 2018). In this way, mutant OPTN and TBK1 can interfere with the process of mitophagy, while mutant p62 shows a lower affinity to LC3-II which leads to impaired mitophagy (Moore and Holzbaur, 2016). These data suggest that the inefficient turnover of damaged mitochondria and also aggregates, may contribute to disease progression in ALS (Weishaupt et al., 2016). In line with the argument that impaired autophagy/mitophagy as a driver of ALS, pharmacological or genetic up-regulation of the SIRT1/NAD^+^-mitophagy axis alleviates disease phenotypes in ALS mice and ALS patients (Blacher et al., 2019; de la Rubia et al., 2019; Lautrup et al., 2019). A detailed summary of defective mitophagy in AD, PD, ALS, and Huntington’s disease is available (review in Lautrup et al., 2019; Lou et al., 2019). In summary, mounting evidence from animals and post-mortem human brain tissues suggests that defective mitophagy is a common feature, and likely plays a causative role in many neurodegenerative pathologies. We summarized the relationships between AD, PD, and ALS, and defective mitophagy/autophagy (Figure 2).

## Mitophagy Inducers

Since reduced mitophagy is common in AD, and maybe a causal mechanism, up-regulating mitophagy might provide a therapeutic strategy for AD (Kingwell, 2019). Small molecules that do not have toxicity to mitochondria (mitochondrial toxicants), but can induce the expression of mitophagy proteins or enhance mitophagy machinery hold translational promise (Ryu et al., 2016; Andreux et al., 2019; Fang, 2019; Lou et al., 2019). The classical mitochondrial uncouplers, e.g., carbonyl cyanide-p-(trifluoromethoxy)phenyl hydrazine (FCCP) and carbonyl cyanide m-chlorophenyl hydrazone (CCCP), and mitochondrial toxins that damage mitochondrial respiration (such as valinomycin, salinomycin, antimycin A and oligomycin) (Georgakopoulos et al., 2017), may have limited translational value for AD because treatment with those drugs will result in dysfunction of normal mitochondria.

In addition, multiple novel mitophagy inducers acting independently of the respiration failure without perturbing the organelle have been reported, offering new momentum to comprehend the process and underlying strategy for therapeutic revolution (Georgakopoulos et al., 2017). One example is to enhance the PINK1/Parkin-mediated mitophagy by supplementation with the ATP analog kinetin triphosphate (KTP) which can amplify catalytic activity of both PD related mutant PINK1^*G*309*D*^ and PINK1^*wt*^ (Hertz et al., 2013) or the application of a p53 inhibitor pifithrin-a, which can release Parkin from binding to the cytosolic p53 in pancreatic β-cells (Hoshino et al., 2014). Moreover, the anti-diabetic natural compound Metformin has been shown to maintain mitochondrial integrity and boost mitochondrial biogenesis through Parkin-mediated mitophagy induction via p53 inhibition (Song et al., 2016; Palikaras et al., 2018). Targeting the up-regulation of the mammalian NF-E2 related factor 2 (Nrf2) (SKN-1, the *C. elegans* ortholog) pathway also enhances mitophagy, with molecules like the compound p62-mediated mitophagy inducer (PMI) (East et al., 2014) and the natural compound Tomatidine affluent in the green tomato (Fang et al., 2017b). NAD^+^ is a fundamental molecule in human health and life since it participates in glycolysis, TCA cycle, OXPHOS, β-oxidation, and many other bioenergetic and metabolic pathways (Verdin, 2015; Fang et al., 2017a; Aman et al., 2018; Mitchell et al., 2018). NAD^+^ is reduced in biological aging, accelerated aging, and in common neurodegenerative diseases, including AD (Mouchiroud et al., 2013; Fang et al., 2014; Hou et al., 2018). Interventional studies support a causative role of NAD^+^ depletion in neurodegeneration, as augmentation of tissue NAD^+^, through the supplementation of nicotinamide riboside (NR) and NMN, can improve neuronal resilience and survival in both premature aging conditions and in AD, through a mitophagy-dependent manner (Fang et al., 2014, 2016a, 2019). Mechanistically, NAD^+^ induces mitophagy through the NAD^+^/Sirtuins-dependent pathways and several other pathways as we summarized elsewhere (Fang, 2019). In conclusion, small molecule which can induce mitophagy *in vivo*, but circumvent the cellular toxicity, hold promise for further clinical studies on AD.

## Future Perspectives

**FIGURE 3:**
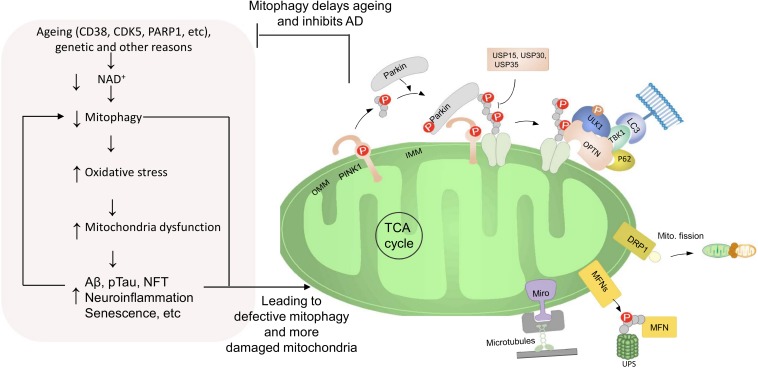
Roles of defective mitophagy in AD with a vicious cycle between them. In normal condition, different mitophagy pathways are activated to efficiently eliminate damaged or superfluous mitochondria. E.g., the PINK1-Parkin pathway is a well-characterized mitochondrial recycling pathway. PINK1 is stabilized on the OMM of a damaged mitochondrion, promoting Parkin recruitment. Parkin ubiquitinates several outer membrane components. Poly-Ub chains are subsequently phosphorylated by PINK1 for the autophagic machinery. Adaptor proteins (e.g., p62, OPTN, NDP52, ULK1, and TBK1) recognize phosphorylated poly-Ub chains on mitochondrial proteins and initiate autophagosome formation through binding with LC3. Furthermore, the PINK1–Parkin pathway modulates mitochondrial dynamics and motility by targeting MFN and Miro for proteasomal degradation. However, aging, genetic, and non-genetic factors cause impairment of the NAD^+^-mitophagy axis, which exacerbates disease defining pathologies of AD, including higher Aβ, pTau, and inflammation. These disease pathologies can further cause the damage of mitochondria and the inhibition of mitophagy, thus, generating a vicious cycle.

Accumulating data suggest the existence of PINK1/Parkin-dependent and –independent mitophagy pathways that are critical in the maintenance of mitochondrial homeostasis as well as neuronal resilience against proteinopathies and stressors. A growing understanding of AD pathology suggests that accumulation of damaged mitochondria due to impaired mitophagy, contributes to Aβ/Tau proteinopathies and inflammation, which may ultimately lead to neuronal loss and memory impairment. Accordingly, experiments from *C. elegans* and mouse models of AD and from AD iPSC-derived neurons suggest that turning up mitophagy might mitigate AD pathologies and retain cognition (in AD animals) with possible mechanisms summarized (Figure 3). Some outstanding questions need to be further addressed. First, whether defective mitophagy is an early event preceding and causing Aβ/Tau proteinopathies? Second, what are the additional molecular mechanisms of defective mitophagy in AD? Cellular signaling and progresses, including histone modification and DNA methylation, DNA repair, senescence, and cell to cell communication (including neurons and glial cells) link to neural plasticity and cognitive function (Halder et al., 2016; Fang et al., 2019; Zhang et al., 2019). Possible linkages of mitophagy in these processes should be explored (Figure 1). Third, whether pharmacological restoration of mitophagy could rescue/delay the progression of memory loss in AD patients? Because no single strategy has been effective in treating AD, it is possible that a multi-targeted combinational approach, and even personalized treatments will be necessary to treating AD. Since long-term multidomain intervention, including intervention of diet, exercise and cognitive training, could improve or maintain cognitive functioning in at-risk elderly people from the general population, a role of mitophagy is worthy of further exploration (Ngandu et al., 2015). The availability of new experimental systems, including the AD patient-oriented neuronal and glial cells (Haenseler et al., 2017; Lin et al., 2018; Volpato et al., 2018), the 3D tri-culture system (Park et al., 2018), and the application of artificial intelligence (Aman et al., 2019) will enable mechanistic studies in models more closely resembling the human AD brain, and will propel drug development.

## Author Contributions

CX and EF designed the outline of the review and wrote the draft of the review. YA, BA, MC, HP-F, and JX provided scientific comments and wrote part of the review.

## Conflict of Interest

EF has CRADA arrangements with ChromaDex, and is a consultant to Aladdin Healthcare Technologies and the Vancouver Dementia Prevention Centre. The remaining authors declare that the research was conducted in the absence of any commercial or financial relationships that could be construed as a potential conflict of interest.
